# The Role of Soy Isoflavones in the Prevention of Bone Loss in Postmenopausal Women: A Systematic Review with Meta-Analysis of Randomized Controlled Trials

**DOI:** 10.3390/jcm11164676

**Published:** 2022-08-10

**Authors:** Agnieszka Barańska, Wiesław Kanadys, Magdalena Bogdan, Ewa Stępień, Bartłomiej Barczyński, Anna Kłak, Anna Augustynowicz, Marta Szajnik, Urszula Religioni

**Affiliations:** 1 Department of Medical Informatics and Statistics with e-Health Lab, Medical University of Lublin, 20-090 Lublin, Poland; 2 Specialistic Medical Center Czechow, 20-848 Lublin, Poland; 3 Department of Social Medicine and Public Health, Warsaw Medical University, 02-007 Warsaw, Poland; 4 Department of Virology with SARS Laboratory, Medical University of Lublin, 20-093 Lublin, Poland; 5 I Chair and Department of Oncological Gynaecology and Gynaecology, Medical University of Lublin, 20-081 Lublin, Poland; 6 Department of Environmental Hazards Prevention, Allergology and Immunology, Warsaw Medical University, 02-091 Warsaw, Poland; 7 Department of Economics of Health and Medical Law, Medical University of Warsaw, 02-091 Warsaw, Poland; 8 Medical Faculty, Lazarski University, 02-662 Warsaw, Poland; 9 School of Public Health, Centre of Postgraduate Medical Education of Warsaw, 01-826 Warsaw, Poland

**Keywords:** soy, bone mass, meta-analysis, postmenopausal woman, bone loss, randomized controlled trials

## Abstract

The aim of the report was to determine the effects of soy isoflavones on lumbar spine, femoral neck, and total hip bone mineral density (BMD) in menopausal women. MEDLINE (PubMed), EMBASE, and Cochrane Library databases were searched for articles published in English during 1995–2019. Studies were identified and reviewed for inclusion and exclusion eligibility. Weighted mean differences (WMD) were calculated for each study and were pooled by using the random effects model. Eighteen randomized controlled trials were selected for meta-analysis. Different types of soy phytoestrogens, i.e., genistein extracts, soy isoflavones extracts, soy protein isolate, and foods containing diverse amounts of isoflavones were used in the studies. The analysis showed that daily intake of 106 (range, 40–300) mg of isoflavones for 6–24 months moderately but statistically significantly positively affects BMD, compared with controls: lumbar spine WMD = 1.63 (95% CI: 0.51 to 2.75)%, *p* = 0004; femoral neck WMD = 1.87 (95% CI: 0.14 to 3.60)%, *p* = 0.034; and total hip WMD = 0.39 (95% CI: 0.08 to 0.69)%, *p* = 0.013. Subgroups analyses indicated that the varying effects of isoflavones on BMD across the trials might be associated with intervention duration, racial diversity (Caucasian, Asian), time after menopause, form of supplements (especially genistein), and dose of isoflavones. Our review and meta-analysis suggest that soy isoflavones are effective in slowing down bone loss after menopause.

## 1. Introduction

Bone remodeling processes allow the bone to adapt to changes in mechanical load, provide repair of microdamages or fractures and are mechanisms for its renewal. Under physiological conditions in bone tissue, there are two dynamic processes, osteolysis and osteogenesis. Although bone mass is mainly genetically determined (70–85% of individual variability), many factors, such as functioning of the endocrine system, physical activity, eating habits, and stimulants affect its quality [1]. Bone mass changes with age.

After peak bone mass is reached by the end of the second decade of life, a period of relative stabilization is observed in the third decade of life. Around the age of 40, there is a gradual decrease in bone mass (0.3–0.5% annually), which is an expression of the beginning of the domination of the resorption process over bone formation [2,3]. In the fifth decade of age is noted an accelerated bone loss. The bone loss increases two years before menopause, reaching the highest level during the first 3 years of post-menopause, losing 3–5% annually, then, after 5–10 years, the slow phase is observed, which lasts indefinitely [4,5].

The postmenopausal decline in estrogen levels is the main or only cause of accelerated bone turnover which results in lower bone mineral density (BMD) and a weakening of the spatial structure of bone tissue. As a consequence, its brittleness and susceptibility to fracture (postmenopausal osteoporosis) is increased [6]. Because bone loss is a result of hypoestrogenism, hormone therapy (HT) is a rational approach in the peri-and postmenopausal period, especially in women with osteopenia. The Women’s Health Initiative (WHI) trial has demonstrated that HT effectively reduces the incidence of all fractures in postmenopausal women, even those with low risk [7,8]. However, the WHI results regarding the increased incidence of adverse events, such as the increase of thromboembolic diseases risk, including stroke and myocardial infarction, as well as breast cancer, marginalized the use of HT [9,10]. Therefore, the search for safer alternatives for the prevention/treatment of osteoporosis is continued.

Among Asian women, fractures associated with osteoporosis occur less often than in Western countries, which allowed stating that one of the possible reasons is the traditional Asian diet rich in phytoestrogens contained in soy protein [11] Asian women consume 25–45 mg of isoflavones per day, compared to 5 mg/d taken by western women [12]. Genistin, daidzin, and glycitin as glycosides and the corresponding forms of aglycones (genistein, daidzen, and glycitein) are the major isoflavones in soy. After ingestion, the glycosidic forms are hydrolysed by the enzymes of the digestive system and its microflora to the aglycone forms in the jejunum [13]. The released aglycones are either absorbed intact by the intestines or are further metabolised by the intestinal flora to other products, including equol [14,15]. Approximately 30–50% of the population is capable of producing it [16,17]. Isoflavones exhibit structural and functional similarity to 17 beta-estradiol. These have a two-dimensional effect on bone metabolism, stimulating bone formation by directly affecting osteoblasts while inhibiting the resorption activity of osteoclasts by activating estrogen receptors [18,19,20].

The aim of the study was a systematic review, with a meta-analysis summarizing clinical randomized controlled trials (RCTs), assessing the effect of soy isoflavones on bone loss in postmenopausal women.

## 2. Materials and Methods

### 2.1. Search Strategy and Study Selection

The selection of the included publications came about through a three-stage process. First, a preliminary search of the bibliographic databases MEDLINE (PubMed), EMBASE, and Cochrane Library from the period between January 1995 and December 2019 occurred. This was based on the following keywords: phytoestrogens “OR” isoflavones “OR” genistein “AND” bone “OR” bone mass “OR” bone mineral density/BMD “OR” osteoporosis. Second, a decision to include or exclude after analyzing the full texts of the selected articles was then made. Third, data was subsequently collected from each qualified work on the clinical and methodological characteristics of the research and test statistics [21].

### 2.2. Inclusion and Exclusion Criteria

The studies selected for the analysis met the following criteria: (a) It included healthy peri- and postmenopausal women, (b) it consisted of a randomized, blinded clinical trial with a parallel control group that were published in English, (c) it involved a research group with a standardized isoflavones extract with a clearly administered dose (tablets, capsules), genistein (Gen) extract (tablets), and isolated soy protein (IBS) in powder form in a mix with beverages, food and snacks, and soy products with varying levels of isoflavones enrichment; (d) the study included a placebo or non-isoflavones control group; (e) the data consisted of BMDs of the lumbar spine (LS), femoral neck (FN), and total hip (TH), and (f) BMD results as measured by dual-energy X-ray absorptiometry (DXA).

### 2.3. Data Extraction

For the purposes of the analysis, the following data were collected from each selected study: first author’s name, year of publication, country in which the study was conducted, study design, sample size, randomization, analysis and exclusion/resignation, initial and final BMD of LS, FN, and TH in each test arm, expressed as the mean change (M), standard deviation (SD), and group size (n). If the standard error of the mean (SEM) was shown in the statistical analysis, it was converted into SD using the standard formula [SEM = SD/√n], and in the 95% confidence interval (95% CI)—by formula [95% CI = SD/√n (SEM) × 1.96]. In the case of two or more active groups (different isoflavones values), compared to one control group, one combined measure was calculated. Moreover, to avoid duplication of data from the same groups with multiple time points, only endpoints with the longest duration were taken into account.

### 2.4. Quality Assessment and Bias Risk of the Trials

The RCT quality was assessed according to the scale developed by Jadad et al. [22] The instrument is a three-item checklist that provides an assessment of bias, specifically, randomization (0 to 2 points), blinding (0 to 2 points), and withdrawals/dropout (0 to 1 point). The quality ranged from 0 to 5 points. Two reviewers independently assessed the studies.

### 2.5. Statistical Analysis and Meta-Analysis

The primary criterium was to determine the overall effect of the administered isoflavones on changes in BMD in relation to the baseline values in three sites of the skeleton using weighted mean difference (WMD) and 95% CI. The mean percentage changes in BMD were compared in the comparison, intervention and control groups, and calculated using the formula: (final data − output data)/output data × 100%.

An additional analysis was undertaken to investigate possible factors that may refer to the potential effects of soy phytoestrogens on postmenopausal studies (≤5 years vs. >5 years <10 vs. ≥10 years), type of supplement (Gen, mixture isoflavones, foods with isoflavones), isoflavones doses (<90 mg, ≥90 mg), racial differentiation (Asian, Caucasian), and duration of intervention (<12 months, 12 months, 24 months). A secondary meta-analysis of the subgroups defined above was performed to estimate the impact of these factors on 10 or more tests in the analysis and 2 or more in each subgroup, comparing the percentage changes. Statistics Q and test I^2^ were used to assess the quantification of heterogeneity and to calculate the proportion of variation due to heterogeneity [23,24]. STATISTICA Medical Software StatSoft (Krakow, Poland) was used for all statistical analyzes.

## 3. Results

### 3.1. Characteristics of Included Trials

**Figure 1:**
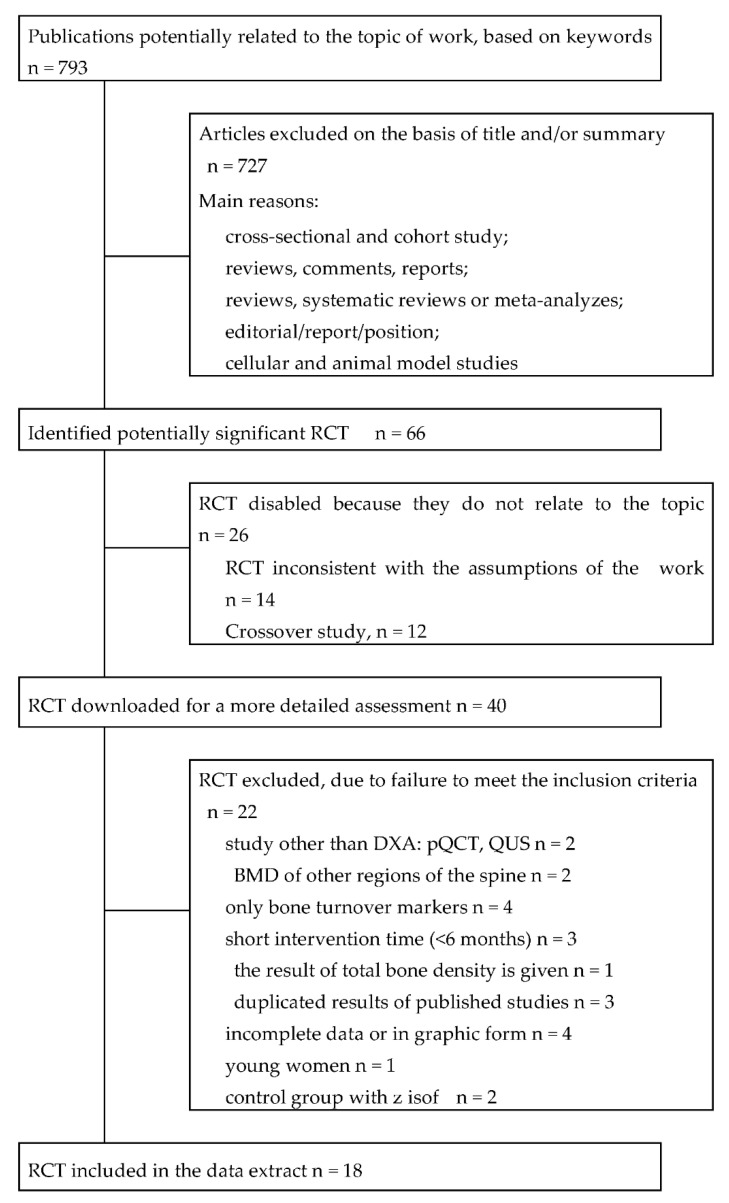
Literature search and research selection process. Abbreviations: RCT, randomized, controlled clinical trial; DXA, dual-energy X-ray absorptiometry; QCT, quantitative computer tomography; QUS, quantitative ultrasonography; BMD, bone mineral density.

Detailed review and selection processes are presented in Figure 1. Eighteen studies evaluating the effects of soy isoflavones on bone mass that met the inclusion criteria and were identified in a systematic review [25,26,27,28,29,30,31,32,33,34,35,36,37,38,39,40,41,42] have been included in the work. Five RCTs were conducted in East Asia, including two in Taiwan, one each in China, Hong Kong, and Japan, eight in the USA, one in Canada, and five in Europe: two in Italy, one each in Denmark and The Netherlands, and a multicenter study (Italy, France, and The Netherlands). The duration of follow-up was from 6 months to 2 years.

The characteristics of the research are summarized in Table 1. The analysis covered postmenopausal women, with the exception of participants in the study of Alekel et al. [26], which were in the perimenopausal period. The mean postmenopausal period was 7.74 ± 5.71 years. A total of 2350 women participated in the studies, including 1260 in the active groups and 1090 in the control groups. The number of individual trials ranged from 43 to 431. The mean age of the participants was 57.0 ± 5.78 years (range, 39–83). The mean body mass index (BMI) equaled 25.28 ± 3.78 kg/m^2^. The studies did not show any significant differences between the research groups in relation to age, postmenopausal years, BMI, and BMD. Isoflavones was administered in the form: extract of Gen, Gen with daidzein, isoflavones extract, soy milk, or IBS with different levels of isoflavones enrichment. The average dose of the supplemented isoflavones was 106.2 ± 60.9 mg/d (median, 90; range, 40–300).

### 3.2. BMD of the Lumbar Spine

**Figure 2:**
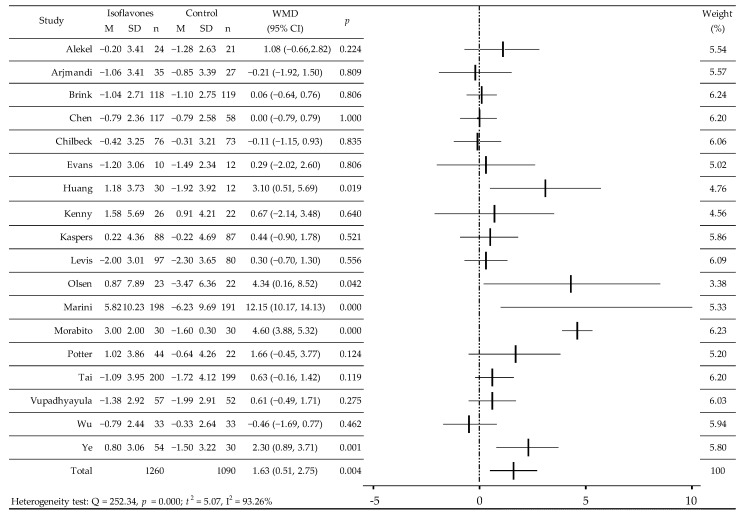
The effect of soy isoflavones on bone mineral density of the lumbar spine in postmenopausal women, compared with placebo [25,26,27,28,29,30,31,32,33,34,35,36,37,38,39,40,41,42]. M, mean change from baseline (%); SD, standard deviation; n, size of the study group; WMD, weighted average difference (%); CI, confidence interval; horizontal lines correspond to 95% CI (some of them go beyond the limits of the scale).

RCT value of BMD was analyzed in 18 RCTs before and after administration of isoflavones or placebo (Table 1) [25,26,27,28,29,30,31,32,33,34,35,36,37,38,39,40,41,42]. There was a significant increase in BMD [27,30,33,34,36] and a slight upward trend [25,29,39] as well as in comparison with the control group, slowing down of bone loss [26,28,35,37,38,40,41] or its reduction [31,32,42]. In the random effects model, the weighted average difference (WMD) was 1.63 (95% CI: 0.51, 2.75)%, *p* = 0.0042; Q = 252.34, *p* = 0.000; t^2^ = 5.07, I^2^ = 93.26% (Figure 2). Additional meta-analysis after excluding two research studies [27,36] with extreme results that could affect the final result confirmed the moderate, statistically significant effect of isoflavones on BMD L1-L4: WMD 0.48 (0.09–0.88)%, *p* = 0.0166, Q = 22.2745, *p* = 0.1008; t^2^ = 0.1895; I^2^ = 32.66%. Meta-analysis of the subgroups showed a statistically significant effect of isoflavones consumption on BMD in Caucasian women, over 5 years after menopause, after 12 and 24 months of observation, regardless of the dose of isoflavones/d, and especially after receiving Gen (Table 2).

### 3.3. BMD of the Femoral Neck

**Figure 3:**
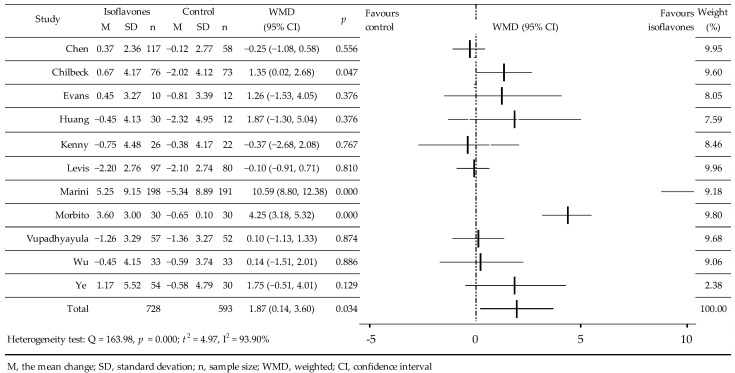
Effect of soy isoflavones on femoral neck bone mineral density in postmenopausal women compared to placebo [27,28,32,33,34,35,36,38,39,40,42]. M, mean change from baseline (%); SD, standard deviation; n, size of the study group; WMD, weighted average difference (%); CI, confidence interval; horizontal lines correspond to 95% CI (some of them go beyond the boundaries of the scale.

In 11 RCTs, the influence of isoflavones on BMD was studied. In these, 1321 women participated, including 728 in the active groups and 593 in the control groups [27,28,32,33,34,35,36,38,39,40,42]. Two studies used IBS enriched with isoflavones [35,39], in the others, standardized isoflavones, and in two of them, Gen [27,36]. In three cases, the isoflavones value [28,33,34] was averaged (Table 1). In four studies, an increase in BMD [27,33,35,36] was observed compared to the control—deceleration of bone loss was noted [32,34,38,42] and three studies saw decrease [28,39,40]. Meta-analysis of the data showed a significant effect of isoflavones on BMD, WMD = 1.87 (95% CI: 0.14, 3.60)%, *p* = 0.0342; Q = 163.98; *p* = 0.000; t^2^ = 4.97, I^2^ = 93.90% (Figure 3). Supplementary meta-analysis after excluding two studies [27,36] showed a small, but statistically non-significant effect on FN, BMD: WMD = 0.21 (−0.25, 0.66)%, *p* = 0.3772; Q = 8.1814, *p* = 0.4160; t^2^ = 0.01185; I^2^ = 2.22%. In the analysis of the subgroups, a significant increase in BMD was observed after the admission of Gen-significant, however, with a borderline level of significance in the western population and following a period of 5–10 years after menopause (Table 2).

In 11 RCTs, the influence of isoflavones on BMD was studied. In these, 1321 women participated, including 728 in the active groups and 593 in the control groups [27,28,32,33,34,35,36,38,39,40,42]. Two studies used IBS enriched with isoflavones [35,39], in the others, standardized isoflavones, and in two of them, Gen [27,36]. In three cases, the isoflavones value [28,33,34] was averaged (Table 1). In four studies, an increase in BMD [27,33,35,36] was observed compared to the control—deceleration of bone loss was noted [32,34,38,42] and three studies saw decrease [28,39,40]. Meta-analysis of the data showed a significant effect of isoflavones on BMD, WMD = 1.87 (95% CI: 0.14, 3.60)%, *p* = 0.0342; Q = 163.98; *p* = 0.000; t^2^ = 4.97, I^2^ = 93.90% (Figure 3). Supplementary meta-analysis after excluding two studies [27,36] showed a small, but statistically non-significant effect on FN, BMD: WMD = 0.21 (−0.25, 0.66)%, *p* = 0.3772; Q = 8.1814, *p* = 0.4160; t^2^ = 0.01185; I^2^ = 2.22%. In the analysis of the subgroups, a significant increase in BMD was observed after the admission of Gen-significant, however, with a borderline level of significance in the western population and following a period of 5–10 years after menopause (Table 2).

### 3.4. BMD of the Total Hip

**Figure 4:**
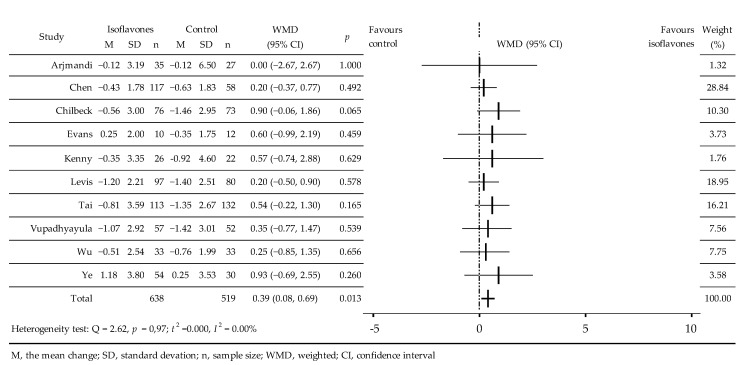
Effect of soy isoflavones on bone mineral density of proximal femur in postmenopausal women compared to placebo [28,31,32,33,35,38,39,40,41,42]. M, mean change from baseline (%); SD, standard deviation; n, size of the study group; WMD, weighted average difference (%); CI, confidence interval; horizontal lines correspond to 95% CI.

The effects of isoflavones on BMD were analyzed in 10 RCTs [28,31,32,33,35,38,39,40,41,42]. A total of 1157 women took part in these, 638 in intervention groups and 519 in control groups, which took soy isoflavones extract [28,32,33,39,40,41,42] or products with IBS enriched with isof [31,35,38], (Table 1). Compared with the control group, a negligible increase in BMD was noted [33,35] as well as a slight slowdown in bone density decrease [28,31,32,38,39,40,41,42]. Meta-analysis revealed a small but significant effect isoflavones on BMD, without statistical heterogeneity, WMD = 0.39 (95% CI: 0.08, 0.69)%, *p* = 0.013; Q = 2.62, *p* = 0.97; t^2^ = 0.00, I^2^ = 0.00% (Figure 4). Analysis of the subgroups showed significant effects of the isoflavones at 90 mg/day, isoflavones mixtures, and after 24 months of observation; borderline significance of BMD increases in the western population and negligible effect of elapse of time from menopause (Table 2).

## 4. Discussion

The analysis of the effects of soy isoflavones on bone mass in individual research studies showed a contradiction in results. Some studies reported an increase of bone mineral density after isoflavones consumption, whereas others have not found any effect. In some, a positive effect appeared in certain examined bone regions of the skeleton, but not in others. Our meta-analysis, however, which included 18 RCTs, generally confirmed the efficacy of isoflavones in slowing down the loss of bone mass caused by estrogen deficiency after menopause.

The literature on the subject contains five meta-analyses of the results of the evaluation of effects of isoflavonoids from soy on the skeleton of postmenopausal women that show opposite results. This may result from the fact that a different number of tests were selected for individual analyses, covering different sizes of the studied female populations, using different inclusion and exclusion criteria. Moreover, the designed interventions used different types of soy isoflavones extracts, including alone genistein and dietary products containing different amounts of phytoestrogens.

Ma et al. [43], based on 10 RCTs of 608 women, found a statistically significant increase in LS BMD compared to placebo (WMD = 20.6 (95% CI: 4.5, 36.6) mg/cm^2^, *p* = 0.01). The meta-analysis of Taku et al. [44], including 11 RCTs, covered data from 1240 women and revealed that the daily intake of an average of 82 (47–150) mg soy isoflavones for 6–12 months significantly increased LS BMD by 22.25 (95% CI: 7.62, 32.89) mg/cm^2^, *p* = 0.002 or 2.38 (95% CI: 0.93, 3.83)%, *p* = 0.001). In addition, FN BMD increased insignificantly by 16.89 (95% CI: −2.34, 36.11) mg/cm^2^, *p* = 0.09 or by 2.45 (95% CI: −0.31, 5, 21)%, *p* = 0.08 (7 RCT, 868 women) and TH BMD by 2.45 (95% CI: −1.41, 0.63) mg/cm^2^, *p* = 0.21 or 0.05 (95% CI: −0.53, 0.63)%, *p* = 0.86 (5 RCT, 420 women) [44]. Lambert et al. [45], in the meta-analysis based on 26 RCTs (2652 women), showed a moderately significant effect of isoflavones on bone loss in estrogen-deficient women (WMD (g/cm^2^) in LS 0.01 (95% CI: 0.01, 0.02), *p* < 0.000 and in TH 0.001 (95%: CI 0.00, 0.02), *p* < 0.01). In contrast to the abovementioned, Liu et al. [46], based on 10 eligible RCTs (986 women), revealed it was unlikely that isoflavones supplementation had a significant beneficial effect on LS BMD 0.4%, FN −0.3%, and TH 0.25%, compared to the control group. In addition, based on 12 RCTs, covering 1433 women of the western population, Ricci et al. [47] also did not observe a statistically significant interaction of isoflavones on BMD in L1-4, WMD = 9.86 (95% CI: −2.64, 22.36) mg/cm^2^.

The mechanism of action of isoflavones increasing bone mass is not fully understood. In animal and in vitro models, isoflavones reveal their biological effects in target cells by means of genomic mechanisms, activating estrogen receptors (ER), with higher affinity towards ER-β [19]. They strengthen the ER by binding to a specific element of the DNA response, the estrogen response element (ERE) [20]. The non-genomic effect is manifested by activation of the estrogen receptor [18]. They also show osteoprotective activity by inhibiting the activity of tyrosine kinases, as well as the expression of the osteoprotegerin gene (OPG) by affecting the RANKL/RANK/OPG system; by stimulating the insulin-like growth factor-1 (IGF-1) gene and influencing the increase of IL-4 and IL-13 synthesing; as well as by reducing IL-6 and TNF-α; and activating receptors for vitamin D3 and peroxisome proliferator-activated receptors (PPARs), particularly PPARα and PPARγ. Moreover, they display antioxidant activity [48,49,50,51,52,53,54,55].

The ambiguity of the results regarding the efficacy of isoflavones in the literature of the subject raises some limitations. The main analysis of bone mineral density concerned the lumbar spine because it was the most commonly described study. Of all the skeletal sites, this is probably the most sensitive area to estrogen-like activity due to the high trabecular bone content [56]. The total bone remodeling cycle takes about 160 days, and many studies have had a short research period of 6 months, which may be insufficient to assess the effects of isoflavones. What is more, changes in BMD may cause short-term remodeling and not long-term effects [57]. Moreover, the short observation period could have had some influence on the obtained result. Bone resorption can be slowed down by short-term consumption of isoflavone, and that periods longer than a year are likely to be needed to affect BMD. Beyond the aforementioned, a significant part of the conducted research was characterized by a small number of participants. In addition, various doses and forms of phytoestrogens were used in studies on this topic: unmodified soy products, isolated soy protein enriched with isoflavones, standardized extract of soybean isoflavones-mixture, or single isoflavones, genistein. The inter-individual differences regarding the bioavailability and metabolism of the administered isoflavones at the same dose should also be considered, and the variability of the response can be expected. Lastly, with relation to isoflavone consumption safety, it seems that they are safe and that the most common adverse effect is mild and occurs at the gastrointestinal level. According to the results gathered in the present review, it can be stated that there is scientific evidence showing the beneficial effect of isoflavones on bone health and thus in the prevention and treatment of osteoporosis on postmenopausal women [58,59,60], although the results do not seem entirely conclusive as there are discrepancies among the studies, probably related to their experimental designs. For this reason, the results should be interpreted with caution, and more randomized clinical trials are required.

## 5. Conclusions

Our work showed a moderate, but statistically significant effect of soy isoflavones on bone mass. Their consumption could provide an important strategy to control bone loss in postmenopausal women, which has some clinical relevance in therapy. Based on the results of our study and the literature of the subject, it should be emphasized that further long-term randomized trials of the effects of phytoestrogen supplementation on bone mass, involving a larger number of postmenopausal women and the use of graduated preparations, are necessary.

## Figures and Tables

**Table 1:** Of the randomized blinded clinical trials with a control group on the influence of soy isoflavones on bone mineral density (listed chronologically).

Author (Year) Country Observation Time	Participants ^a^ Age, y ^b^ Inclusion Criterion	Test Report		Average Output Values BMD (g/cm^2^)	Jadad Scale
		Active Group	Control Group		
Potter [25] 1998 USA —6 months	66/66 (–%) 60.8 ± 8.6 (39–83) 2.8 ± 8.8 ysm, BMI 27.8 ± 5.3	IFa 90 mg isof/d IFb 55.6 mg isof/d 40 g SP	Con, 40 g casein and non-fat milk powder	LS: IFa 0.892; IFb 0.971; Con 0.940	5
Alekel [26] 2000 USA —6 months	69/69 (–%) 49.8 (44–59) 1.6 ysm, BMI 24.1 ± 3.4	IF 80.4 mg isof-ag/d 40 g SP	Con, 40 g whey protein	LS: IF 0.981; Con 1.000	5
Morabito [27] 2002 Italy —12 months	90/90 (–%) 51.5 ± 3.1 (47–57) 6.5 ± 2.5 ysm, BMI 24 ± 3, FN BMD T-score > −1 SD	IF tabl 54 mg Gen/d (purity ~98%)	Pla tabl, nd.	LS: IF 0.915; Pla 0.934 FN: IF 0.687; Pla 0.689	3 ^c,e^
Chen [28] 2003 Hong Kong —12 months	203/175 (13.8%) 54.3 ± 3.3 (48–62) 4.1 ± 2.4 ysm, BMI 24.0 ± 3.5	IFa caps, 80 mg isof-ag/d IFb caps, 40 mg isof-ag/d (46.4% Dai, 38.8% Gly, 14.7% Gen)	Pla caps, corn starch	LS: IFa 0.860; IFb 0.874; Pla 0.846 FN: IFa 0.680; IFb 0.688; Pla 0.679 TH: IFa 0.809; IFb 0.824; Pla 0.823	5
Kaspers [29] 2004 The Netherlands —12 months	202/175 (13.4%) 66.6 ± 4.8 (60–75) 17.9 ± 6.9 ysm, BMI 26.1 ± 3.8	IF 99 mg isof-ag/d (53% Gen, 41% Dai, 6% Gly), 25.6 g SP	Con, 25.6 g milk protein	LS: IF 0.917; Con 0.895	5
Olsen [30] 2004 Denmark —2 years	107/89 (16.8%) 57.1 ± 7.6 (<75) 10.9 ysm, BMI 23.9 ± 3.9, LS BMD T-score < −1.0 SD	IF 76 mg isof-ag/d, 17.5 g SP	Con, 17.5 g SP without isof	LS: IF 0.865; Con 0.835	4 ^d^
Arjmandi [31] 2005 USA —12 months	87/62 (28.7%) 54.5 ± 5.0 (<65) 5.5 ± 5.0 ysm, BMI 27.9 ± 7.4	IF 60 mg isof/d, 25 g SP	Con, 25 g SP without isof	LS: IF 0.944; Con 0.941 TH: IF 0.853; Con 0.871	4 ^c^
Wu [32] 2006 Japan —6 months	136/128 (5.9%) 54.4 ± 2.9 (45–60) 3.2 ± 1.8 ysm, BMI 21.1 ± 2.4	IF caps 75 mg isof/d, (47 mg isof-ag)	Plac caps, dextrin	LS: IF 0.891; Pla 0.907 FN: IF 0.668; Pla 0.676 TH: IF 0.777; Pla 0.787	4 ^c^
Ye [33] 2006 China —6 months	90/84 (6.7%) 52.3 ± 3.3 (45–60) 2.6 ± 1.5 ysm, BMI < 30	IFa caps, 126 mg isof-ag/d, IFb caps, 84 mg isof-ag/d (52% Dai, 15% Gen, 33% Dai)	Pla caps, starch	LS: IFa 0.892; IFb 0.839; Pla 0.864 FN: IFa 0.725; IFb 0.692; Pla 0.690 TH: IFa 0.813; IFb 0.796; Pla 0.792	5
Huang [34] 2006 Taiwan —12 months	43/42 (2.3%) 52.3 ± 2.5 (45–67) 4.4 ± 1.1 ysm, BMI 23.5 ± 3.6	IFa 200 mg isof-ag/d, IFb 100 mg isof-ag/d (71% Gen, 29% Dai)	Pla tabl, nd.	LS: IFa 1.07; IFb 1.09; Pla 1.06 FN: IFa 0.85; IFb 0.80; Pla 0.78	2 ^c,d,e^
Evans [35] 2007 USA —9 months	61/43 (29.5%) 64.7 ± 5.1 (50–65) 8.3 ± 5.1 ysm, BMI 26.8 ± 2.4	IF 91.5 mg isof-ag/d, 25.6 g SP	Con, 25.6 g milk protein	LS: IF 0.915, Con 0.939 FN: IF 0.673, Con 0.738 TH: IF 0.801, Con 0.857	4 ^d^
Marini [36] 2007 Italy —2 years	389/389 (–%) 54.5 ± 3.1 (49–67) 5.3 ± 3.5 ysm, BMI 25.1 ± 3.8, FN BMD T-score < –1.0	IF tabl, 54 mg Gen/d (czystość > 98%)	Pla tabl, calcium carbonate	LS: IF 8.842, Pla 0.837 FN: IF 0.667, Pla 0.674	5
Brink [37] 2008 Italy, France, The Netherlands —12 months	300/237 (21.0%) 53 ± 3 2.7 ± 1.3 ysm, BMI 24.5 ± 2.1, LS BMD T-score > −2	IF 110 mg isof-ag/d (60–75% Gen, 25–35% Dai, 1–5% Gly), biscuits, bars	Con, biscuits, cereal bars	LS: IF 0.983, Con 0.995	4 ^c^
Vupadhyayula [38] 2009 USA —2 years	203/157 (22.7%) 63.5 ± 4.5 (>55) 14.3 ± 5.4 ysm, BMI 26.3 ± 3.8, LS BMD T-score > −2.5	IF 90 mg isof/d, 25 g SP	Con, 25 g milk protein	LS: IF 1.085, Con 1.104 FN: IF 0.873, Con 0.881 TH: IF 0.931, Con 0.910	5
Kenny [39] 2009 USA —12 months	131/97 (25.9%) 72.6 ± 5.9 (>60) 23.3 ± 9.5 ysm, BMI 28.1 ± 5.1, LS BMD T-score > −3.0	IF tabl, 105 mg isof-ag/d (Gen, Gly, Dai)	Pla tabl, maltodextrin	LS: IF 1.140, Pla 1.103 FN: IF 0.804, Pla 0.795 TH: IF 0.860, Pla 0.866	5
Levis [40] 2011 USA —2 years	248/177 (28.6%) 52.5 ± 3.3 (45–60) 5.5 ± 0.5 ysm, BMI 26.3 ± 3.3, LS BMD T-score ≥ −2.0	IF tabl, 200 mg isof/d	Pla tabl, nd.	LS: IF 1.146, Pla 1.132 FN: IF 0.940, Pla 0.937 TH: IF 0.990, Pla 0.982	5
Tai [41] 2012 Taiwan —2 years	431/399 (7.4%) 55.9 ± 3.8 (43–65) 5.1 ± 2.7 ysm, BMI 22.9 ± 2.5, LS BMD T-score > −1.0	IF caps, 300 mg isof-ag/d (57.5% Gen, 42.5% Dai)	Pla caps, microcrystalline cellulose, xylitol	LS: IF 0.863, Pla 0.866 TH: IF 0.813, Pla 0.775	5
Chilibeck [42] 2013 Canada —2 years	351/298 (15,1%) 56.5 ± 6.8 5.0 ± 2.6 ysm, BMI 27.4 ± 4.1, LS BMD T-score > −2.51	IF tabl, 165 mg isof/d (105 mg isof-ag: Gen, Dai and Gly in a ratio of 1:1:0.2)	Pla tabl, dicalcium phosphate, magnesium stearate, sorbitol	LS: IF 0.951, Pla 0.958 FN: IF 0.746, Pla 0.741 TH: IF 0.887, Pla 0.890	5

Data are means ± standard deviation. Abbreviations: BMD, bone mineral density (g/cm^2^); BMI, body mass index (kg/m^2^); caps, capsule; Con, control group; d, daily/day; Dai, daidzein; FN, femoral neck; Gen, genistein; Gly, glycitein; IF, active group; isof, isoflavones; isof-ag, isoflavones in the form of a glycons; LS, lumbar spine; nd., no data; Pla, placebo; SP, soy proteim; tabl, tablet, TH, total hip; T-score, the BMD of the subject to the average BMD of the young person; ysm, years since menopause (y). ^a^ sample size: randomisation/analysis (exclusion indicator), ^b^ range, ^c^ deduct one point because the method of randomization was described, but was inappropriate, ^d^ deduct one point because the method of blinding was described, but was inappropriate, ^e^ deduct one point because of no description of withdrawal and dropouts.

**Table 2:** Analysis in subgroups of the effect of soy isoflavones on bone mineral density in postmenopausal women.

	Lumbar Spine BMD				Femoral Neck BMD				Total Hip BMD			
Variables	n	N	WMD (95% CI), *p*	Q Test, *p*	n	N	WMD (95% CI), *p*	Q Test, *p*	n	N	WMD (95% CI), *p*	Q Test, *p*
Overall												
	18	2350	1.63 (0.51; 2.75), 0.004	252.34, 0.000	11	1321	1.87 (0.14; 3.60), 0.034	163.98, 0,000	10	1157	0.39 (0.08; 0.69), 0.013	2.62, 0.97
Populations												
Asian	5	766	0.80 (−0.18; 1.77), 0.111	14.1296, 0.0069	4	367	0.32 (−1.08; 1.30), 0.515	3.9466, 0.2674	4	590	0.35 (−0.06; 0.76), 0.092	1.0289, 0.7943
Western	13	1584	1.91 (0.35; 3,47), 0.017	14.1296, 0.0069	7	954	2.45 (−0.09; 4.99), 0.059	144.1531, 0.0000	6	567	0.44 (−0.02; 0.91), 0.061	1.4452, 0.9192
Years since menopause												
≤5 years	7	798	0.52 (−0.20; 1.24), 0.161	16.0434, 0.0135	5	516	0.64 (−0.29; 1.57), 0.178	6.5691, 0.1605	4	474	0.40 (−0.03; 0.83), 0.068	2.0062, 0.5711
5–10 years	6	1109	2.93 (0.14; 5.71), 0.039	176.8500, 0.0000	4	648	4.00 (−0.38; 8.39), 0.073	128.3974, 0.0000	4	526	0.37 (−0.12; 0.85), 0.136	0.5698, 0.9033
≥10 years	5	443	0.81 (0.07; 1.56), 0.033	3.7921, 0.4349	2	157	0.01 (−1.10; 1.11), 0.993	0.1128, 0.7369	2	157	0.39 (−0.61; 1.40), 0.445	0.0282, 0.8667
Period of observation												
<12 months	5	283	0.95 (−0.20; 2.10), 0.106	9.2890, 0.0543	3	172	0.91 (−0.39; 2.20), 0.169	1.2182, 0.5438	3	172	0.50 (−0.29; 1.29), 0.216	0.4841; 0.7850
12 months	7	799	1.04 (0.00; 2.07), 0.049	84.5048, 0.0000	4	325	1.40 (−1.43; 4.24), 0.332	44.2279, 0.0000	3	285	0.21 (−0.33; 0.75), 0.446	0.1145; 0.9444
24 months	6	1268	2.79 (0.24; 5.34), 0.032	131.8048, 0.0000	4	824	1.92 (−2.34; 6.19), 0.377	146.2043, 0.0000	4	700	0.46 (0.04; 0.88), 0.032	1.4181; 0.7013
Type of supplement												
genistein	3	491	6.63 (1.61; 11.64), 0.010	52.3530, 0.0000	3	491	5.67 (0.87; 10.47), 0.021	41.1603, 0.0000				
Isof extract	7	1098	0.37 (−0.19; 0.94), 0.197	11.0512, 0.0868	6	699	0.22 (−0.39; 0.82), 0.486	6.4699, 0.2631	7	964	0.39 (0.06; 0.72), 0.0216	2.4611; 0.8728
Isof food	8	761	0.40 (−0.07; 0.87), 0.094	6.9289, 0.4363	2	131	0.29 (−0.84; 1.42), 0.615	0.5555, 0.4561	3	193	0.39 (−0.48; 1.25), 0.379	0.1542; 0.9258
The dose of isof												
<90 mg/d	9	966	2.69 (0.44; 4.93), 0.019	198.2354; 0.0000	5	748	3.07 (−0.59; 6.72), 0.100	137.7879; 0.0000	4	361	0.22 (−0.26; 0.70), 0.377	0.0646; 0.9957
≥90 mg/d	9	1384	0.58 (0.08; 1.08), 0.022	14.4621; 0.1068	6	573	0.53 (−0.18; 1.24), 0.143	7.7546; 0.2566	6	796	0.47 (0.07; 0.88), 0.020	1.4899; 0.9602

Abbreviations: isof, isoflavones; n, number of studies; N, number of women; WMD, weighted mean difference (%); CI, confidence interval.

## Data Availability

Not applicable.
